# Elevated kindlin-2 promotes tumour progression and angiogenesis through the mTOR/VEGFA pathway in melanoma

**DOI:** 10.18632/aging.102187

**Published:** 2019-08-19

**Authors:** Chuan-Yuan Wei, Meng-Xuan Zhu, Peng-Fei Zhang, Xuan Yang, Lu Wang, Jiang-Hui Ying, Wen-Jie Luan, Cheng Chen, Jia-Qi Liu, Ming Zhu, Yan-wen Yang, Zi-Hao Feng, Fa-Zhi Qi, Jian-Ying Gu

**Affiliations:** 1 Department of Plastic Surgery, Zhongshan Hospital, Fudan University, Shanghai 200032, P.R. China; 2 Department of Liver Surgery and Transplantation, Liver Cancer Institute and Zhongshan Hospital, and Key Laboratory of Carcinogenesis and Cancer Invasion, Ministry of Education, Fudan University, Shanghai 200032, P.R. China; 3 Liver Cancer Institute, Zhongshan Hospital, Fudan University, Key Laboratory of Carcinogenesis and Cancer Invasion, Fudan University, Ministry of Education, Shanghai 200032, P.R. China; 4 Department of Oncology, Shanghai East Hospital, Tongji University School of Medicine, Shanghai 200032, P.R. China

**Keywords:** kindlin-2, melanoma, mTOR/VEGFA, angiogenesis, prognosis

## Abstract

Background: In our previous study, kindlin-2 promoted skin wound healing and decreased the permeability of neovascularization during angiogenesis. Herein, we explored the biological function and underlying mechanism of kindlin-2 in cutaneous melanoma.

Methods and Results: Through a series of *in vitro* assays, we found that high levels of kindlin-2 promoted migration and invasion of melanoma cells without influencing cell proliferation. Quantitative real-time polymerase chain reaction (qRT-PCR) and western blot analyses showed that upregulated kindlin-2 promoted the cellular epithelial-mesenchymal transition (EMT). Importantly, we found that melanoma cells overexpressing kindlin-2 promoted angiogenesis and VEGFA secretion *in vitro* and facilitated tumour growth and lung metastasis *in vivo*. To unveil the underlying mechanism, we conducted Next-generation sequencing (NGS) and differential expression analyses. Kyoto Encyclopedia of Genes and Genomes (KEGG) analysis showed that overlapping differentially expressed genes (DEGs) were primarily enriched in the TGF-β, mTOR and VEGF signalling pathways. Then, we confirmed that the mTOR/VEGFA pathway was activated during the process of kindlin-2-induced melanoma progression and angiogenesis. Moreover, we demonstrated that kindlin-2 was significantly overexpressed in clinical melanoma samples and that a high level of kindlin-2 predicted a poor prognosis.

Conclusions: Taken together, these findings showed that kindlin-2 promotes angiogenesis and tumour progression via the mTOR/VEGFA pathway.

## INTRODUCTION

Melanoma is an aggressive form of cutaneous tumours and causes 55,500 deaths annually [1]. Sustaining proliferation signalling and activating invasion and metastasis are primary features of melanoma [2]. For example, activating mutations in the MAPK pathway, which incorporates the enzymes RAS, RAF, MEK and ERK, result in constitutive signalling, leading to melanoma cell proliferation and apoptosis resistance [3]. In order to progress, tumour cells require sustained nutrients, oxygen and the ability to excrete metabolic wastes, and the tumour-associated neovasculature plays a crucial role in this process [4]. Multiple factors are involved in the promotion and maintenance of angiogenesis in melanoma, such as growth factors, receptors, cytokines and other cellular components [5]. Among them, vascular endothelial growth factor A (VEGFA) exerts an important role in endothelial cell proliferation and vascular remodelling by activating its tyrosine kinase receptor (VEGFR) in melanoma [6]. In view of this, bevacizumab, a humanized monoclonal antibody that neutralizes the VEGFA isoforms, is one of the most frequently investigated antiangiogenic molecules with a certain curative effect in melanoma [7–8].

Kindlins are FERM (four-point-one, ezrin, radixin, moesin) domain proteins comprising three members (kindlin-1, -2 and -3), which are evolutionarily conserved [9]. Among them, kindlin-2, encoded by the *FERMT2* gene (chromosome 14q22.1), is the most broadly distributed [10]. Kindlin-2 has emerged as a key activator of integrin through binding to the cytoplasmic tails of the integrin β subunit [11]. Kindlin-2 plays various biological functions under physiological conditions, such as cell migration, adhesion, spreading and organization of the actin cytoskeleton [12]. A deficiency in kindlin-2 results in early embryonic lethality of mice, which provides powerful evidence of its important role [13–14]. Recently, aberrant kindlin-2 has been reported to be involved in a variety of malignancies. Shen and colleagues showed that kindlin-2 promotes the invasion of gastric cancer cells through the phosphorylation of integrin β1 and β3 cytoplasmic domains [15]. Zhan et al. showed that kindlin-2 is upregulated by TGF-β signalling and that TGF-β1-induced kindlin-2 in turn upregulates TGFBR1, thereby providing positive feedback to drive the progression of pancreatic ductal adenocarcinoma (PDAC) [16]. In addition, kindlin-2 expression in tumour cells also plays an important role in the interaction between host cells and the tumour microenvironment (TME), as it regulates the expression of CSF-1 and EGF, which is required for autocrine and paracrine crosstalk between cancer cells and macrophages, and then promotes tumour growth [17]. However, whether kindlin-2 plays a role in melanoma remains unknown.

In this research, we conducted a series of experiments to explore the biological function and underlying mechanism of kindlin-2 both in *in vivo* and *in vitro* assays. Furthermore, the relationship between its expression and clinical significance was analyzed via a large number of melanoma samples. Our results will help further understand the molecular mechanisms underlying melanoma progression and identify potential therapeutic targets for melanoma.

## RESULTS

### Elevated kindlin-2 promotes melanoma invasion and migration

**Figure 1:**
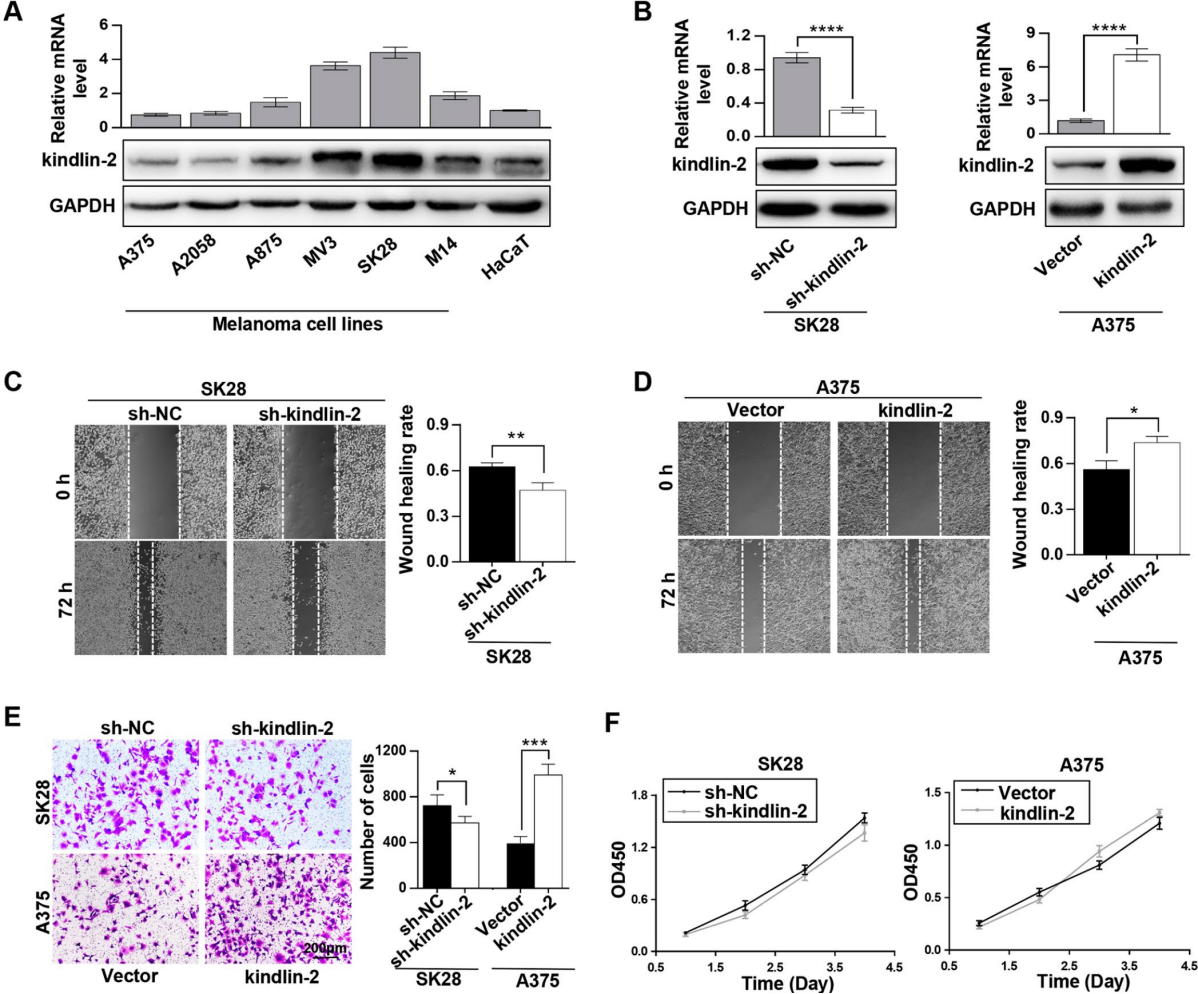
**Elevated kindlin-2 promotes melanoma cell invasion and migration.** (**A**) The mRNA and protein levels of kindlin-2 in six melanoma cell lines and HaCaT, a normal skin cell line. (**B**) The efficiencies of kindlin-2 knockdown and overexpression were examined by western blotting and qRT-PCR. (**C** and **D**) The migration ability of SK28-shNC and SK28-sh-kindlin-2 (**C**), A375-Vector and A375-kindlin-2 (**D**) cells was measured by wound-healing assays. (**E**) Invasion ability was measured in the indicated cells by Matrigel invasion assays. (**F**) The effects of kindlin-2 knockdown and overexpression on proliferation ability were measured by CCK-8 assays. ^*^p<0.05, ^**^p<0.01, ^***^p<0.001,^****^p<0.0001.

The recognized role of kindlin-2 was examined first. Through qRT-PCR and western blotting analyses, we found that kindlin-2 was differentially expressed in different melanoma cell lines, which showed a highly heterogeneity of melanoma. Among them, kindlin-2 was overexpressed in four of six melanoma cell lines compared with HaCaT, a normal skin cell line (Figure 1A). Then, the short hairpin RNA (shRNA) lentivirus vector of kindlin-2 was transfected into SK28 cells, and the kindlin-2 cDNA vector was transfected into A375 cells (Figure 1B). Through wound-healing migration and Matrigel invasion assays, we found that high levels of kindlin-2 promoted migration and invasion, while low levels of kindlin-2 inhibited migration and invasion of melanoma cells (Figure 1C–1E). However, we found that kindlin-2 had no influence on the proliferation of melanoma cells analyzed by CCK-8 assays (Figure 1F). Together, we found that high levels of kindlin-2 promote cellular invasion and migration in melanoma.

### High levels of kindlin-2 induce the cellular EMT

**Figure 2:**
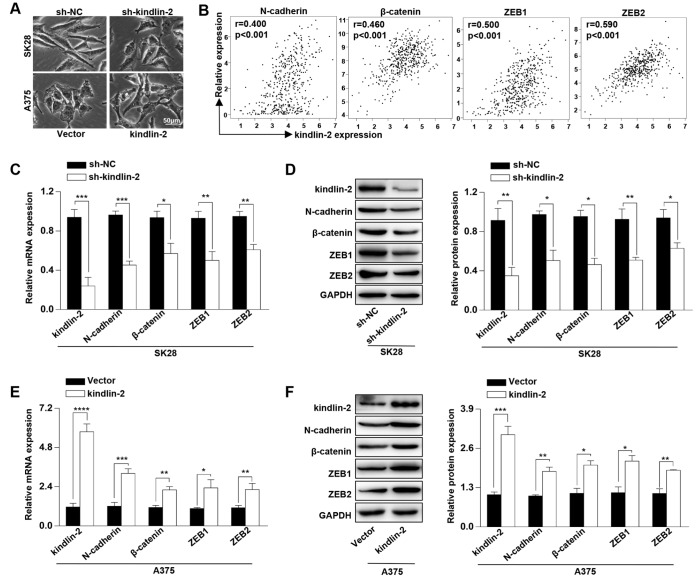
**High levels of kindlin-2 induce the cellular EMT in melanoma.** (**A**) The cell morphology of SK28-shNC, SK28-sh-kindlin-2, A375-Vector, and A375-kindlin-2 cells was observed by a phase-contrast microscopy. (**B**) Correlation analyses between the expression of kindlin-2 and N-cadherin, β-catenin, ZEB1, ZEB2 were performed with the TCGA database. (**C** and **E**) The mRNA levels of N-cadherin, β-catenin, ZEB1 and ZEB2 in SK28-shNC, SK28-sh-kindlin-2, A375-Vector and A375-kindlin-2 cells were analyzed by qRT-PCR. (**D** and **F**) The protein levels of N-cadherin, β-catenin, ZEB1 and ZEB2 in the indicated cells were analyzed by western blotting. ^*^p<0.05, ^**^p<0.01, ^***^p<0.001.

Kindlin-2 has been reported to induce the tumour cellular EMT [18–19]. Through a phase-contrast microscopy, we found that SK28-shNC and A375-kindlin-2 cells presented spindle-like, fibroblastic morphologies compared with SK28-sh-kindlin-2 and A375-Vector cells, which joined closely like epithelial cells (Figure 2A). Then we performed correlation analyses between the expression of kindlin-2 and EMT markers through the TCGA database, and found a positive correlation between kindlin-2 and N-cadherin (p < 0.001, r = 0.400), β-catenin (p < 0.001, r = 0.460), ZEB1 (p < 0.001, r = 0.500), and ZEB2 (p < 0.001, r = 0.590) (Figure 2B). Through qRT-PCR and western blot analyses, we confirmed that high levels of kindlin-2 increased, while low levels of kindlin-2 decreased the mRNA and protein levels of N-cadherin, β-catenin, ZEB1 and ZEB2 (Figure 2C–1F). These results show that elevated kindlin-2 promotes the cellular EMT in melanoma.

### High levels of kindlin-2 facilitate vascular formation and the secretion of VEGFA

**Figure 3:**
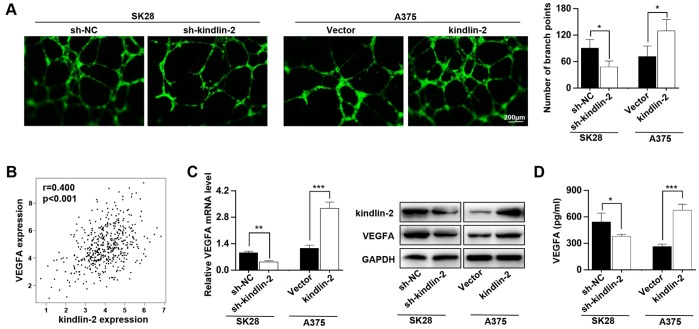
**High levels of kindlin-2 promote angiogenesis and VEGFA secretion in melanoma cells.** (**A**) The tube formation assays were performed with the conditioned medium of SK28-shNC, SK28-sh-kindlin-2, A375-Vector and A375-kindlin-2 cells. (**B**) The correlation analysis between the expression of kindlin-2 and VEGFA was performed with the TCGA database. (**C**) The mRNA and protein levels of VEGFA in the indicated cells wer analyzed by qRT-PCR and western blotting. (**D**) The levels of VEGFA secreted by the indicated cells were analysed by ELISA. ^*^p<0.05, ^**^p<0.01, ^***^p<0.001.

Our previous study showed that kindlin-2 affects neovascular permeability [20], and here, we detected whether its expression in melanoma cells had an effect on angiogenesis. We found that kindlin-2 overexpression in melanoma cells promoted vascular formation and that the downregulation of kindlin-2 inhibited vascular formation (Figure 3A). As the secretory protein VEGFA plays an important role in angiogenesis, we examined the relationship between the expression of kindlin-2 and VEGFA through TCGA database, and a positive correlation was observed (p < 0.001, r = 0.400, Figure 3B). Through qRT-PCR, western blot and ELISA analyses, we further showed that high levels of kindlin-2 increased the expression and secretion of VEGFA (Figure 3C, 3D). Thus, we conclude that high levels of kindlin-2 promote the secretion of VEGFA and vascular formation.

### Upregulated kindlin-2 induces tumour progression *in vivo*

**Figure 4:**
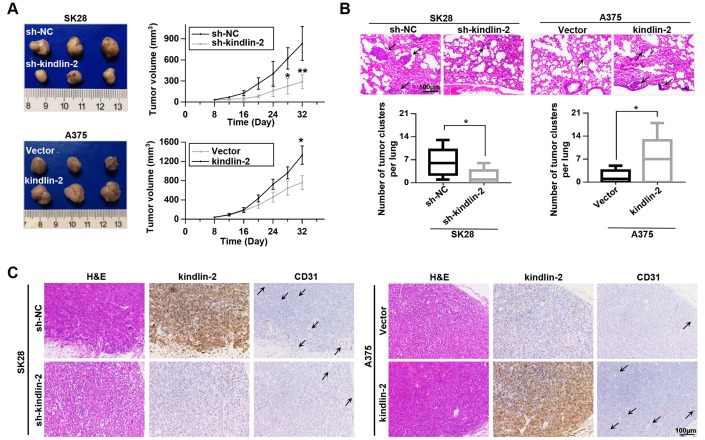
**Kindlin-2 promotes tumour growth and lung metastasis *in vivo*.** (**A**) SK28-shNC, SK28-sh-kindlin-2, A375-Vector and A375-kindlin-2 cells were subcutaneously inoculated to examine tumour growth ability (left); tumour growth curves of the subcutaneous xenografts were shown (right). (**B**) Lung metastasis was investigated with the metastasis model *in vivo*, and the number of metastases was examined by H&E staining. (**C**) Representative images from tumour serial sections stained with H&E and with kindlin-2 and CD31 antibodies by IHC. ^*^p<0.05, ^**^p<0.01.

Since angiogenesis plays an important role in tumour progression [21], we constructed subcutaneous tumourigenesis and lung metastasis models to examine the role of kindlin-2 in tumour progression. Through the subcutaneous tumourigenesis model, we found that high levels of kindlin-2 promoted tumour growth, while low levels of kindlin-2 inhibited tumour growth (Figure 4A). Similarly, kindlin-2 over-expression increased, while kindlin-2 knockdown inhibited lung metastasis examined by a lung metastasis model (Figure 4B). Additionally, IHC staining on the serial sections showed that subcutaneous tumours with overexpressed kindlin-2 had increased vascular density and vice versa, which further confirmed that melanoma cells with kindlin-2 upregulation promotes tumoural angiogenesis. (Figure 4C). Thus, we showed that upregulated kindlin-2 promotes melanoma progression *in vivo*.

### Next-generation sequencing and bioinformatics analyses

**Figure 5:**
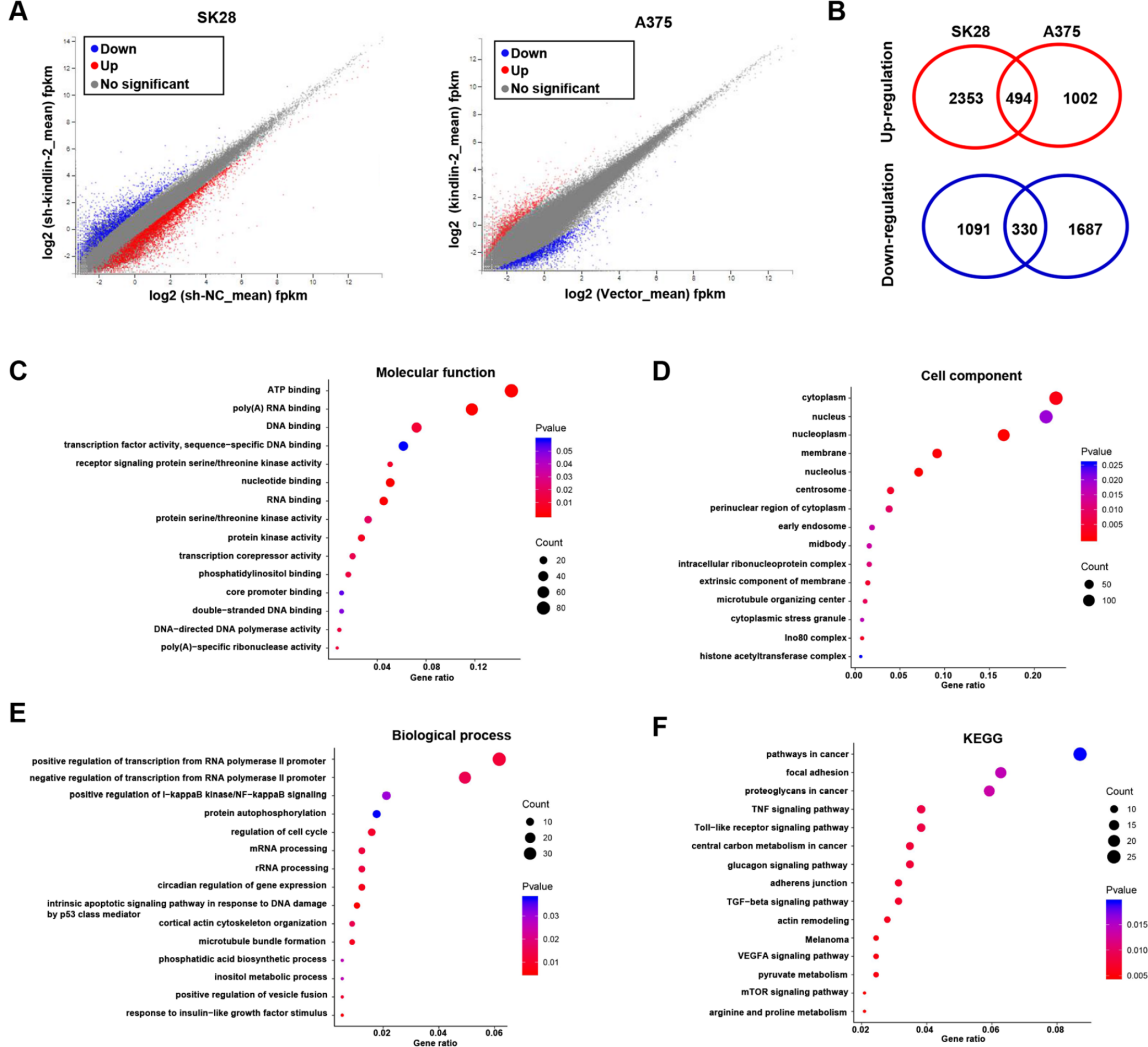
**RNA sequencing and bioinformatics analyses.** (**A**) Differentially expressed genes in SK28-shNC/SK28-sh-kindlin-2 and A375-Vector/A375-kindlin-2 cells were showed by volcano plots. (**B**) Venn diagrams were constructed to show the overlapping upregulated (red) and downregulated (blue) DEGs. (**C**–**F**) Gene Ontology (Molecular function, cell component and biological process) and KEGG analyses of the overlapping DEGs were performed.

To unveil the underlying mechanism of kindlin-2 induced melanoma progression, we performed the gene expression profiles according to the differentially expressed kindlin-2 (Figure 5A). There were 2847 upregulated DEGs and 1421 downregulated DEGs in SK28-shNC cells compared with SK28-sh-kindlin-2 cells, while there were 1496 upregulated DEGs and 2017 downregulated DEGs in A375-kindlin-2 cells compared with A375-Vector cells. Among them, 824 DEGs overlapped, including 494 upregulated DEGs and 330 downregulated DEGs (Figure 5B). According to Gene Ontology (GO) analysis, the overlapped DEGs were classified into three groups: biological process (BP), molecular function (MF), and cellular component (CC). The main MF categories that were enriched included ATP binding, polyA RNA binding, DNA binding, and receptor signalling protein serine/threonine kinase activity (Figure 5C). Within the CC group, the overlapping DEGs were mainly involved in the cytoplasm, nucleus and nucleoplasm (Figure 5D). For BP, the overlapping DEGs were enriched in both the positive and negative regulation of transcription from the RNA polymerase II promoter, the positive regulation of I−κB kinase/NF−κB signalling and protein autophosphorylation (Figure 5E). Additionally, KEGG analysis revealed that the overlapping DEGs were involved in pathways in cancer, focal adhesion, proteoglycans in cancer, TGF-β signalling pathway and so on, all of which are known functions of kindlin-2 (Figure 5F). Interestingly, we also found that the overlapping DEGs were enriched in mTOR and VEGFA signalling pathways, which have not been reported and deserve further verification.

### Kindlin-2 promotes angiogenesis and tumour progression via the mTOR pathway

**Figure 6:**
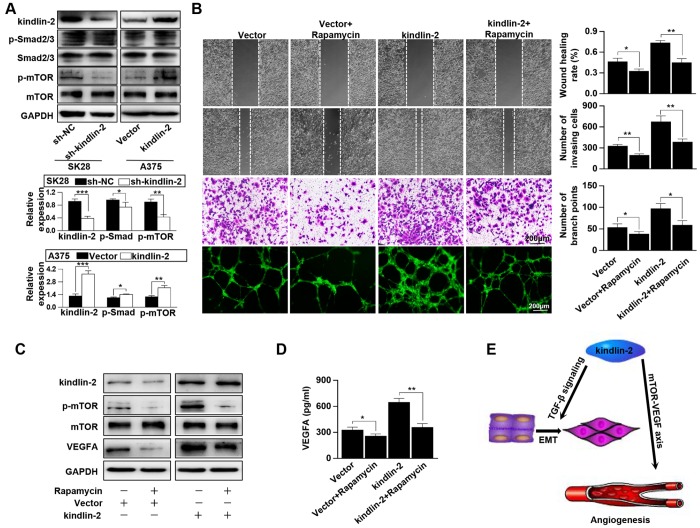
**Kindlin-2 promotes angiogenesis and tumour progression via the mTOR pathway.** (**A**) The protein levels of kindlin-2, p-Smad2/3, Smad2/3, p-mTOR and mTOR were detected in SK28-shNC, SK28-sh-kindlin-2, A375-Vector and A375-kindlin-2 cells by western blotting. (**B**) Migration, invasion and tube formation assays were performed in A375-Vector and A375-kindlin-2 cells after incubation with rapamycin. (**C**) Western blotting was used to detect the levels of kindlin-2, p-mTOR, mTOR and VEGFA in A375-Vector and A375-kindlin-2 cells after incubation with rapamycin. (**D**) ELISA was used to detect the secretion of VEGFA in the indicated cells. (**E**) A schematic model of kindlin-2-induced melanoma progression. ^*^p<0.05, ^**^p<0.01.

According to the results of KEGG, we performed western blotting and found that high levels of kindlin-2 slightly upregulated, while low levels of kindlin-2 slightly downregulated the p-Smad2/3 level. The results indicated that over-expressed kindlin-2 activates the TGF-β pathway, which confirmed the reliability of the NGS results. What’s more, we detected that high level of kindlin-2 increased the p-mTOR level, and that low level of kindlin-2 decreased its level (Figure 6A). Through specific inhibitor, we found that kindlin-2-induced migration, invasion, and vascular formation were reversed by rapamycin (mTOR pathway inhibitor, 100 nM, 24 h, Figure 6B). Furthermore, we found that kindlin-2-induced high levels of p-mTOR and VEGFA secretion were also reversed by rapamycin (Figure 6C, 6D). Together, we conclude that kindlin-2 promotes angiogenesis and tumour progression via mTOR signalling (Figure 6E).

### Kindlin-2 is overexpressed in melanoma, and elevated kindlin-2 predicts a poor prognosis

**Figure 7:**
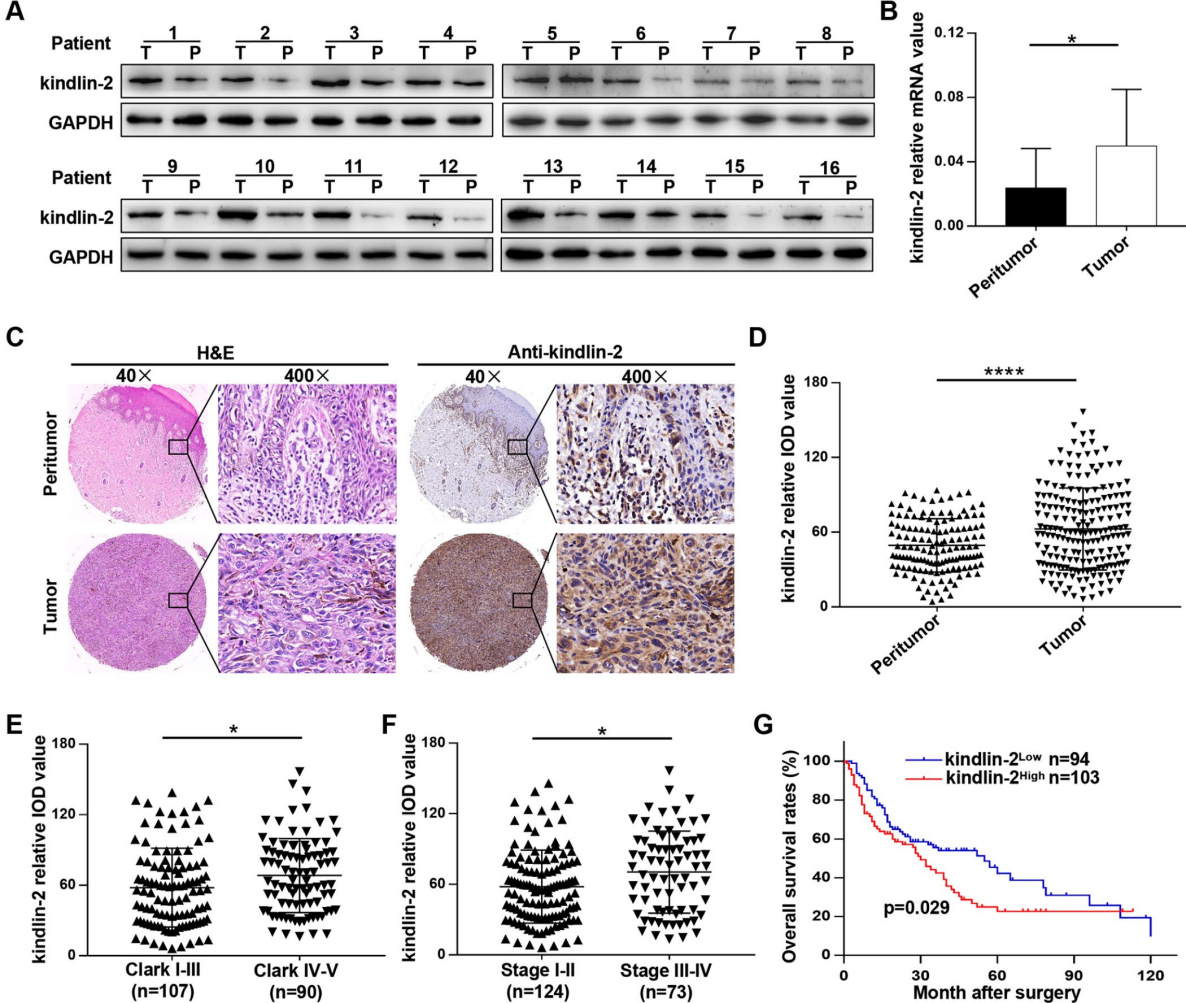
**Kindlin-2 is overexpressed in melanoma, and elevated kindlin-2 predicts a poor prognosis.** (**A**) The protein levels of kindlin-2 in 16 pairs of melanoma and matched peri-tumour tissues. (**B**) The mRNA levels of kindlin-2 in 16 pairs of melanoma and matched peri-tumour tissues. (**C**) Representative images of the TMA after H&E staining and IHC staining with the kindlin-2 antibody. (**D**) The kindlin-2 expression levels in melanoma and peritumoural tissues were analysed by average densitometry. (**E** and **F**) The kindlin-2 levels in different groups were analyzed according to the Clark level and clinical stage. (**G**) Overall survival analysis of 197 melanoma patients according to the expression of kindlin-2. ^*^p<0.05, ^****^p<0.0001. T, tumour; P, peritumour.

Clinically, we found that kindlin-2 was overexpressed in tumour tissues compared with peritumour tissues in sixteen melanoma patients at both mRNA and protein levels (Figure 7A, 7B). Typical IHC images indicated that kindlin-2 was mainly expressed in the cytoplasm but partially expressed in the nucleus (Figure 7C). Through quantification, we further confirmed that kindlin-2 was overexpressed in melanoma tissues compared with paired normal tissues (Figure 7D). Then, patients were divided into two groups according to the Clark level and clinical stage. We found that high kindlin-2 levels were more common in melanoma tissues with advanced Clark levels (p=0.027, Figure 7E) and clinical stages (p=0.011, Figure 7F). Importantly, patients in the kindlin-2^High^ group (n=103) had poorer overall survival rates than those in the kindlin-2^Low^ group (n=94) (p=0.029, Figure 7G). Thus, kindlin-2 is overexpressed in melanoma tissues, and high levels of kindlin-2 predict poor prognoses.

## DISCUSSION

In this study, we explored the potential role and underlying mechanism of kindlin-2 in melanoma. Kindlin-2 promotes migration, invasion and the cellular EMT, and activates the TGF-β pathway. Kindlin-2 also facilitates VEGFA-dependent angiogenesis and tumour progression through the mTOR pathway. Clinically, we show that kindlin-2 is significantly overexpressed in melanoma tissues and predicts a poor prognosis.

Kindlin-2 has been reported to be overexpressed in multiple tumours, and aberrant kindlin-2 expression has been linked to tumour progression. Guo et al. [22] showed that kindlin-2 interacts with and stabilizes EGFR and is required for EGF-induced breast cancer migration, and Ou et al. [23] showed that kindlin-2 promotes glioma cell motility and proliferation both *in vivo* and *in vitro*. In our study, we further confirmed that the overexpression of kindlin-2 as a tumour promoter. Through a series of experiments, we found that elevated kindlin-2 promotes invasion and migration, while there was no influence on proliferation *in vitro*. Furthermore, we found that high levels of kindlin-2 promoted the cellular EMT and that TGF-β signalling was activated during this process.

Previously, we indicated that neovascular permeability was increased and blood vessels were shorter and thinner in kindlin-2 gene knockdown mice [20]. Liao et al. [24] showed that kindlin-2 is necessary for angiogenic sprouting *in vitro* and for tumour angiogenic development *in vivo*. However, these results were based on knocking down kindlin-2 expression in vascular endothelial cells. We were the first to demonstrate the role of tumour-expressed kindlin-2 in the interaction between tumour cells and vascular endothelial cells. We showed that elevated kindlin-2 promoted angiogenesis both *in vivo* and *in vitro*, which well explained that kindlin-2 promoted tumour growth *in vivo* (there was no influence on the proliferation ability *in vitro*). Mechanistically, melanoma cells with high levels of kindlin-2 promoted the secretion of VEGFA, a predominant inducer of both normal and pathophysiological angiogenesis. Furthermore, we analysed the gene expression profiles induced by differentially expressed kindlin-2. KEGG analysis revealed that overlapping DEGs were enriched in the mTOR and VEGFA signalling pathways. A central role of mTOR in the regulation of VEGF expression has previously been shown in multiple cancers [25]. We suggest that elevated kindlin-2 promotes VEGFA-dependent angiogenesis and tumour progression via the mTOR pathway. This is the first report to show that kindlin-2 could promote VEGFA secretion through the mTOR pathway and that this signalling pathway connects tumour cells with vascular endothelial cells.

## CONCLUSIONS

Taken together, our findings confirmed the important role of kindlin-2 in tumour progression and TGF-β pathway activation. Additionally, we provide a new pathway of tumour-expressed kindlin-2 in the communication between tumour cells and vascular endothelial cells. Our study offers novel evidence that kindlin-2 may serve as a potential therapeutic target for melanoma patients.

## MATERIALS AND METHODS

### Patients and follow-up

A tissue microarray (TMA) containing 197 melanoma tissues and 138 normal tissues was used for immunohistochemistry (IHC) analysis. Sixteen melanoma and paired normal tissues were consecutively collected from melanoma patients who underwent complete curative resection for qRT-PCR and western blot analyses. All tissues were collected immediately upon tumour resection, transported in liquid nitrogen and then stored at −80°C. All patients who underwent curative resection were verified by pathological examination at Zhongshan Hospital of Fudan University (Shanghai, China). Clinicopathological information was collected from January 1, 2008, to December 31, 2017. Ethical approval for human subjects was obtained from the Research Ethics Committee of Zhongshan Hospital. Informed consent for collecting and preserving samples and details was obtained from each patient. The overall survival (OS) rate was defined as the interval between surgery and death or the last observation point.

### Cell culture and transfection

HUVECs, HaCaT cells and six melanoma cell lines, namely, A2058, A375, A875, MV3, M14 and sk-mel-28 (shown as SK28), were purchased from a cell bank at the Chinese Academy of Sciences (Shanghai, China). These cells were cultured in Dulbecco’s Modified Eagle Medium (DMEM) or RPMI-1640 medium (HyClone, USA) containing 10% foetal bovine serum (FBS, Invitrogen, USA). The pLKD-CMV-G&PR-U6-shRNA vector and the pLenti-EF1a-mcherry-P2A-Puro-CMV-MCS-3Flag vector were purchased from Obio Technology Corp. (Shanghai, China). The pLKD-CMV-G&PR-U6-shRNA lentiviral vector was transfected into SK28 cells (target sequence GCCTCAAGCTCTTCTT GAT), and the pLKD-CMV-G&PR-U6 vector was used as a negative control. The pLenti-EF1a-mcherry-P2A-Puro-CMV-FERMT2-3Flag vector was transfected into A375 cells, and the pLenti-EF1a-mcherry-P2A-Puro-CMV-MCS-3Flag vector was used as a negative control. The transfection efficiency was examined by western blotting and qRT-PCR.

### Immunohistochemistry

IHC staining was performed as described previously [26]. Briefly, sections that adhered to slides were deparaffinized with xylene and rehydrated with alcohol. After submerging into EDTA antigenic retrieval buffer, diluted hydrogen peroxide (0.3%) was used to inactivate endogenous peroxidase activity, followed by incubation with 5% bovine serum albumin (BSA) and the primary antibody (listed in Supplementary Table 2) overnight at 4°C. Then, the sections were stained with a horseradish peroxidase (HRP)-labelled secondary antibody (Gene Tech, Shanghai, China) and diaminobenzidine (DAB, Gene Tech). Finally, the slide was counterstained with haematoxylin, dehydrated in ethanol, cleared in xylene, and coverslipped. IHC scoring was performed by Image-Pro Plus v6.0 software (Media Cybernetics, Inc., Bethesda, MD).

### qRT-PCR and western blot analyses

Total RNA was extracted from both tissues and cultured cells using TRIzol reagent (Invitrogen, Carlsbad, USA) and reverse-transcribed into cDNA with a PrimeScript RT Reagent Kit (TaKaRa, Japan) according to the manufacturer’s instructions. SYBR Green Real-time PCR Master Mix (Yeasen, Shanghai, China) was used for qRT-PCR analyses carried out in an ABI 7500 Real-Time PCR system (Applied Biosystems, Foster City, USA). The primer sequences for qRT-PCR are shown in Supplementary Table 1. Western blot was performed as described in a previous study [27], and all the primary antibodies are listed in Supplementary Table 2.

### Matrigel invasion, wound-healing migration, CCK-8 and tube formation assays

An invasion assay was performed using a 24-well Transwell plate (8 μm pore size, Corning, NY, USA). Cells (1 × 10^4^) in serum-free medium (200 μl) were seeded in the upper chamber, which was coated with Matrigel (BD Biosciences, USA). Then, 600 μl of complete medium was added to the lower chamber as a chemoattractant. After incubation for 48 h, the membrane was washed briefly with PBS, fixed with 4% paraformaldehyde, stained with crystal violet and counted under a microscope. For the wound-healing migration assay, scratch wounds were produced by a sterile 200 μl pipette tip after washing with cold PBS. The average distance migrated by the cells was measured using a microscope calibrated with an ocular micrometer at a suitable time. For the CCK-8 assay, cells were inoculated into 96-well plates at a density of 1,000 cells per well. Then, 10 μl of CCK-8 reagent (Yeasen, Shanghai, China) was added to the well after the 1^st^, 2^nd^, 3^rd^ and 4^th^ days. The plates were incubated for 2 h, and the absorbance was determined at 490 nm. For tube formation assay, Matrigel was added to a 96-well plate and incubated at 37°C for one hour. Then, HUVECs (1.5 × 10^4^) were seeded into each well with conditioned medium for 6 h. Images were recorded with an Olympus fluorescence microscope.

### Enzyme-linked immunosorbent assay (ELISA)

Cells were plated at 1 × 10^5^ cells per well in a 12-well plate in complete medium overnight, washed with PBS and cultured for another 24 h in 500 μl of serum-free medium. The supernatant was collected and centrifuged to remove cell debris. The secreted VEGFA was quantified by ELISA using the human VEGFA immunoassay kit according to the manufacturer’s protocols (Thermo Fisher Scientific, Carlsbad, CA, USA).

### *In vivo* assay

BALB/c-nu/nu athymic nude mice (4-5 weeks old, 18-20 g) were obtained from the Shanghai Institute of Material Medicine and raised in a specific pathogen-free (SPF) animal laboratory. For the metastasis model, cells (2×10^6^) were injected into the tail veins, and the mice were euthanized after four weeks. The lungs were obtained and fixed with 4% paraformaldehyde. Then, consecutive tissue sections were obtained and stained with haematoxylin-eosin (H&E), and the number of metastatic nodules was counted under a microscope. For the growth model, cells (2×10^6^) were injected into the right flank to generate subcutaneous tumours. Tumour size was measured every four days, and tumour volume was calculated as (length x width^2^)/2. Thirty-two days after injection, the tumour specimens were surgically removed, fixed, paraffin-embedded, and sectioned. The sections were used for H&E and IHC staining. All experimental procedures were approved by the Animal Care Committee of Fudan University (Shanghai, China).

### Statistical analysis

All statistical analyses were carried out using the IBM SPSS Statistics 21 (IBM Corp., USA) statistical software package and GraphPad Prism software V 5.0 (La Jolla, CA, USA). All *in vitro* experiments were repeated at least three times, and the values are presented as the mean ± standard deviation (SD). Student’s t test or Tukey’s multiple comparisons test was used for comparisons between two groups, and one-way ANOVA was used for multiple group comparisons. The survival curve was plotted using the Kaplan-Meier method and compared by the log-rank test. All p values were two-sided, and differences were considered statistically significant at p<0.05.

### Ethics approval

This study was approved by the Ethics Committee of the Zhongshan Hospital Biomedical Research Department and written consent was obtained from all participants.

## Supplementary Material

Supplementary Tables
