# Supplementary material for: Elevated kindlin-2 promotes tumour progression and angiogenesis through the mTOR/VEGFA pathway in melanoma
Source: Aging (Albany NY). 2019 Aug 19;11(16):6273–85. doi: 10.18632/aging.102187 (PMC6738412; doi:10.18632/aging.102187)
Supplement: Supplementary Tables [file aging-11-102187-s001.pdf]

SUPPLEMENTARY TABLES

Supplementary Table 1. Sequences of Primers used for Real-time Polymerase Chain Reaction.

**kindlin-2**	
Forward	5′-CAGACACCCCGAAGAACTTTC-3′
Reverse	5′-GCCCCTCTAATTCAAGTGCCT-3′
**N-cadherin**	
Forward	5′-AGCCAACCTTAACTGAGGAGT-3′
Reverse	5′-GGCAAGTTGATTGGAGGGATG-3′
**β-catenin**	
Forward	5′-AGCTTCCAGACACGCTATCAT-3′
Reverse	5′-CGGTACAACGAGCTGTTTCTAC-3′
**ZEB1**	
Forward	5′-TTACACCTTTGCATACAGAACCC-3′
Reverse	5′-TTTACGATTACACCCAGACTGC-3′
**ZEB2**	
Forward	5′-GCGATGGTCATGCAGTCAG-3′
Reverse	5′-CAGGTGGCAGGTCATTTTCTT-3′
**GAPDH**	
Forward	5′-GGTATGACAACGAATTTGGC-3′
Reverse	5′-GAGCACAGGGTACTTTATTG-3′

Supplementary Table 2. List of Primary Antibodies Used in the Study.

Antibody	Applications	Company
kindlin-2	WB, IF, IP	Abcam (ab74030)
GAPDH	WB, IF, IHC,	Abcam (ab8245)
N-cadherin	WB, IF, IHC, IP	CST (13116)
β-catenin	WB, F, IF, IHC, IP, IHC	CST (8480)
ZEB1	WB, F, IF, IHC	Abcam (ab203829)
ZEB2	WB, IHC	Abcam (ab138222)
p-Smad2/3	WB, IHC	Abcam (ab38449)
Smad2/3	WB, IHC, ELISA,	Abcam (ab8805)
CD31	WB, IHC, IF, F	Abcam (ab32457)
p-mTOR	WB, IF, ELISA	CST (5536)
mTOR	WB, IHC, IF, F	CST (2983)
VEGFA	WB, ELISA	Abcam (ab46154)

Abbreviations: WB: western blot; IHC: immunohistochemistry; IF: immunofluorescence; IP: immunoprecipitation; ELISA: enzyme-linked immunosorbent assay; F: flow cytometric analysis.
